# Aneurysm-related ischemic ventricular tachycardia: safety and efficacy of catheter ablation

**DOI:** 10.1097/MD.0000000000006442

**Published:** 2017-03-31

**Authors:** Jin-Rui Guo, Li-Hui Zheng, Ling-Min Wu, Li-Gang Ding, Yan Yao

**Affiliations:** Fuwai Hospital, Chinese Academy of Medical Sciences and Peking Union Medical College, Beijing, China.

**Keywords:** ablation, complication, efficacy, ventricular aneurysm, ventricular tachycardia

## Abstract

Left ventricular aneurysm (LVA) postmyocardial infarction (MI) might be an arrhythmogenic substrate. We examined the safety and efficacy of catheter ablation of LVA-related ventricular tachycardia (VT).

Thirty-three consecutive patients who underwent primary catheter ablation of ischemic VT were divided into LVA group (11 patients, mean age 61.9 years, 10 men) and none LVA group. Acute procedural outcomes, complications, and long-term outcomes were assessed.

In LVA group, average number of induced VTs were 3.2 ± 2.6 (range 1–7), clinical VTs were located in the ventricular septum scar zone in 4 (36.4%) patients, acute success was achieved in 7 (63.6%) patients, partial success in 3 (27.3%) and failure in 1 patient, while none LVA group showing a statistically similar distribution of acute procedural outcomes (*P* = 0.52). There were no major or life-threatening complications. VT-free survival rate at median 19 (1–44) months follow-up was numerically but not significantly lower in LVA versus none LVA group (48.5% vs 62.8%, log-rank *P* = 0.40).

Catheter ablation of ischemic VT in the presence of LVA appears feasible and effective, with about one-third of cases having septal ablation targets. Further studies are warranted.

## 1 Introduction

Even with rapid myocardial reperfusion after myocardial infarction (MI), left ventricular aneurysm (LVA) may develop, leading commonly to ventricular arrhythmias, congestive heart failure, and thromboembolism.^[1,2]^ Patients with LVA have poor prognosis, with an overall 5-year mortality of 50% ^[3]^ and ventricular arrhythmia as the leading cause of death.^[4]^

Surgery is considered an effective treatment for LVA; however, it has been associated with a high incidence of late sudden death, with malignant ventricular arrhythmia documented after LVA repair in up to 36.8% of fatal cases.^[5]^ Myocardial incision may cause additional tissue damage, possibly impairing cardiac function and increasing surgical risk, especially among patients with reduced ejection fraction.^[6]^

Radiofrequency catheter ablation (RFCA) is commonly used in combination with an implantable cardioverter-defibrillator (ICD) and/or antiarrhythmic therapy for scar-related ventricular tachycardia (VT) associated with structural heart disease.^[7–9]^ However, catheter ablation of VT in patients with ischemic cardiomyopathy can be challenging in the setting of LVA because of the thin apical aneurismal wall consisting almost entirely of hyaline fibrosis tissue. There were a few reports suggesting that RFCA may be a suitable treatment strategy for selected LVA patients with ventricular arrhythmias,^[10–13]^ the safety and long-term efficacy of RFCA in this setting are unknown, and therefore the subject of the present study.

## 2 Methods

### 2.1 Study population

The patient population included 33 consecutive patients, referred for the primary procedure of catheter ablation on documented ischemic VT. All patients provided written informed consent and underwent ablation of VTs at our institutions between January 2013 and February 2016. The diagnosis of ischemic cardiomyopathy was established by history of infarction with Q waves; angiographic evidence such as regional wall motional abnormality and fixed perfusion defect correlating with coronary stenosis; or prior coronary intervention. Aneurysm was defined as a thin-walled bulging left ventricular contour during both diastole and systole.^[14,15]^ The ventricular aneurysms were diagnosed according to the electrocardiogram, left ventriculography, magnetic resonance imaging (MRI), or transthoracic echocardiography.^[16]^

Patients were recruited for the study if they fulfilled the following criteria: documented sustained monomorphic VT with 1 or more episodes in the 6 months before enrollment and at least 2 months post the last ischemic event; failure of at least 2 antiarrhythmic agents. Ablation was considered only after exhausting all other medical options and after detailed risk counseling with informed consent from the patients and their families; and at least 1 clinical VT was induced in the procedure of electrophysiological study. The patients were categorized into 2 groups: LVA group and none LVA group. The study was approved by the local ethical research committee. Informed consent was obtained from all patients.

### 2.2 Electrophysiological study and radiofrequency ablation

Ablation was aimed at transecting the critical VT isthmus or to isolate the scar tissue from the rest of the myocardium thus leading to extensive circumferential ablation patterns in some other cases.^[17,18]^ Mapping and ablation of was performed using 3D electroanatomic mapping with CARTO (Biosense Webster, Diamond Bar, CA) or NavX (Ensite, St. Jude Medical, Minnetonka, MN).

Endocardial left ventricular mapping was performed as previously described.^[19]^ Briefly, ventricular stimulation study was performed with up to 3 extrastimuli to induce VT. The morphologies of the induced VTs were noted. Detailed substrate mapping of the chambers of interest was obtained in all patients. Three-dimensional bipolar electroanatomic maps were displayed with dense scar defined as <0.5 mV, scar border zone from 0.50 to 1.50 mV, and total low-voltage area as >1.5 mV.^[20]^ The baseline voltage cutoffs were then adjusted until all channels were identified.^[21]^ Late potentials in the scar were tagged.

In patients with hemodynamically tolerated VT, activation mapping was performed using 12-lead ECG as reference to guide targeted ablation. Entrainment mapping and pace mapping were also performed. If the VTs were hemodynamically unstable or not inducible but recorded by 12-lead ECG before the EPS procedure, ablation was guided on the basis of detailed characterization of the substrate defined by voltage mapping in sinus rhythm, including identification of split or late potentials, discrete higher voltage channels in the low-voltage region, and/or pace mapping in which the QRS with pacing mimics induced and/or spontaneous VT.^[22]^ During left ventricular endocardial mapping, heparin was given to maintain a target activated clotting time of 200 to 250 seconds.

In all cases, after mapping was completed, RF energy was delivered via the open irrigation catheter at the selected locations based on mapping findings. The power setting parameters were 43°C and up to 40 W. After ablation, patients were monitored for up to 15 minutes for VT recurrence. Ventricular stimulation was performed with up to 3 extrastimuli with and without isoproterenol infusion in all patients after ablation.

### 2.3 Clinical follow-up and outcomes

Outcomes of the study were acute procedural success, long-term procedural success, and safety. Complete procedural success was defined as noninducibility of any VTs that were induced before ablation without or after administration of isoproterenol. Partial success was defined as elimination of only the clinical VT(s). Inducibility of clinical VT(s) at the end of the procedures was considered as procedural failure.

Follow-up after hospital discharge was conducted during outpatient clinic visits every 6 months or whenever an arrhythmic event occurred. Patients were questioned about symptoms, and underwent 24 hours of Holter monitoring during outpatient clinic visits. In all patients with an ICD, the memory of the device was used to detect VTs and to judge procedural success/failure at follow-up. Long-term success was defined as survival and absence of any spontaneous sustained VT during post-procedural follow-up.

Safety was assessed as rate of procedure-related major adverse events (AEs) in 7 days post-ablation. AEs included cardiac perforation, pericardial effusion with hemodynamic compromise, pulmonary embolus, complete heart block, stroke, worsening heart failure, new acute severe mitral or aortic regurgitation, deep venous thrombosis, arterial dissection, injury that required surgical treatment, and death.

### 2.4 Statistical analysis

Descriptive statistics are reported as mean ± standard deviation (SD; or median and range for skewed distributions) for continuous variables, and as absolute frequencies and percentages for categorical variables. Between-group comparisons were performed with the unpaired Student *t* test, the Mann–Whitney *U*-test, or Fisher exact test, as appropriate. VT-free survival after the procedure was evaluated by means of Kaplan–Meier estimation (differences between strata were assessed by the log-rank test). All tests were 2-sided, and *P* < 0.05 was considered statistically significant. Statistical analyses were done using the SPSS 16.0 statistical package (SPSS Inc., Chicago, IL).

## 3 Results

### 3.1 Patient characteristics

The clinical characteristics of the study population are presented in Table 1. The mean age was 59.1 ± 10.4 years, 29 (87.9%) were men, 16 (48.5%) had hypertension, 11 (33.3%) had diabetes mellitus, 4 (11.1%) had chronic kidney disease (CKD), 4 (11.1%) had a history of atrial fibrillation (AF), 15 (45.5%) had a history of percutaneous coronary intervention (PCI), 4 (12.1%) had a history of coronary artery bypass graft (CABG), and 10 (30.3%) had an ICD. Angiographic examination was available in 8 (72.7%) LVA patients and 20 (90.9%) patients without LVA, and no significant stenosis (>50%) was found in these subjects.

**Table 1:**
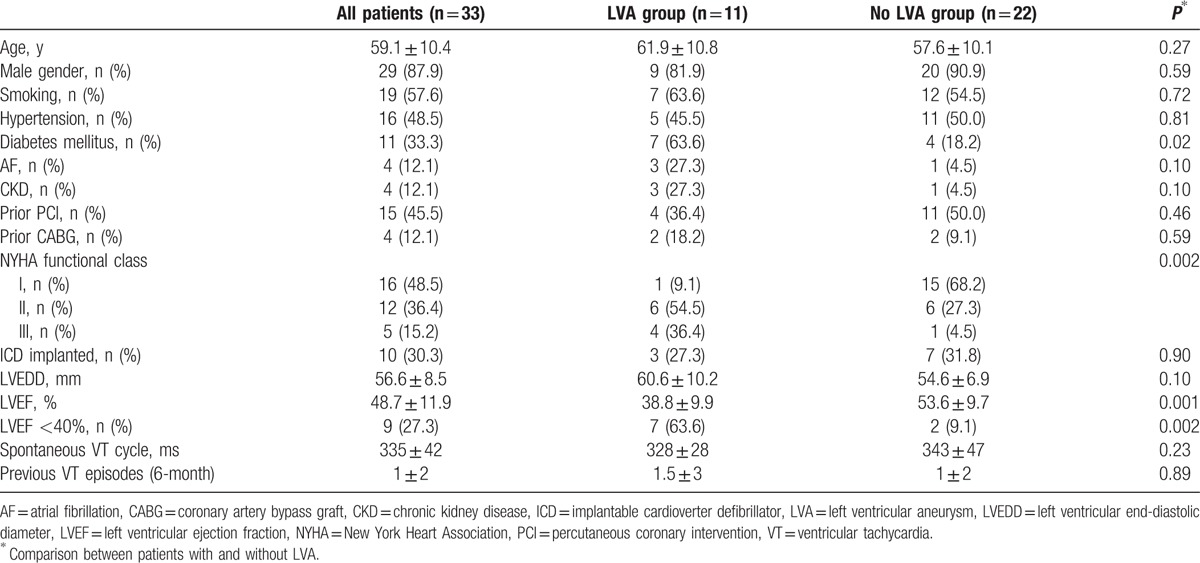
Baseline demographic characteristics of patients overall and by subgroups.

**Figure 1:**
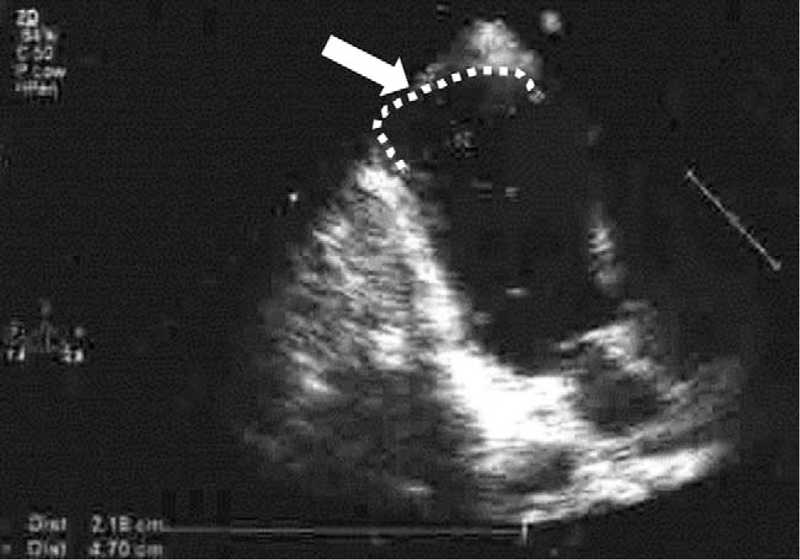
Two-dimensional echocardiographic image in apical in patient #5, 2-chamber view showing a sizable apical aneurysm (arrow).

LVA was found on echocardiogram in 11 (33.3%) patients, and confirmed by late gadolinium-enhanced cardiac MRI or contrast left ventriculogram (Fig. 1). LVA location was apical in all 11 patients of the study cohort. Mean duration of known LVA presence before ablation was 12 ± 7 years (range 3–27 years).

As summarized in Table 1, patients with LVA had lower left ventricular ejection fraction (38.8 ± 9.9 vs.53.6 ± 9.7, *P* = 0.001) with more advanced congestive heart failure [New York Heart Association (NYHA) functional class II/III 90.9% vs 31.8%, *P* = 0.002], and had numerically but not significantly higher proportions of history of CKD (27.3% vs 4.5%, *P* = 0.10) or AF (27.3% vs 4.5%, *P* = 0.10) when compared with the patients without LVA. There were no differences in the other clinical variables between groups.

Details of the clinical characteristics of the patients with LVA are presented in Table 2. The mean age was 61.9 ± 10.8 years, and all but 2 patients were male. Three patients presented with electrical storm, 7 with recurrent VT, and another with incessant VT.

**Table 2:**
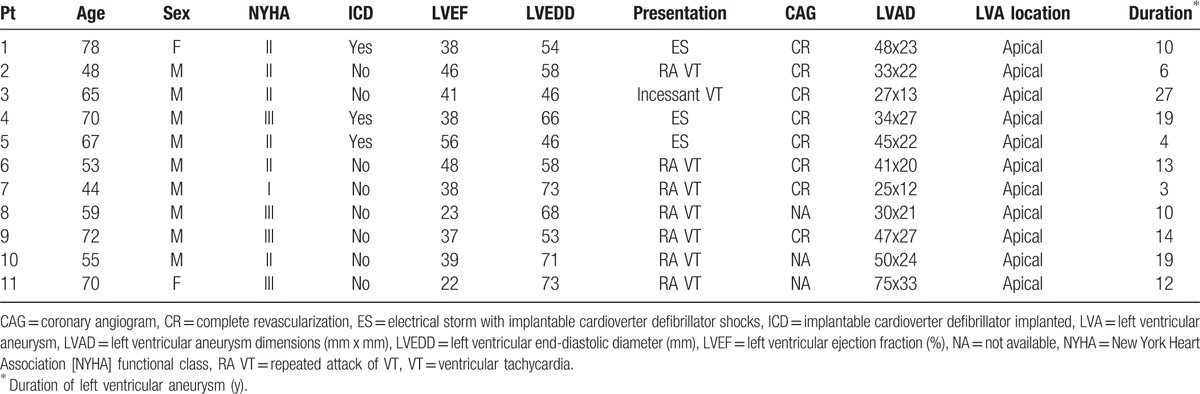
Clinical characteristics of patients presenting with left ventricular aneurysm.

### 3.2 Procedural characteristics and acute outcomes

All patients underwent mapping and ablation in only the endocardium (endo). In the LVA group, 7 patients underwent mapping and ablation in LV, 1 in RV only, and 2 in both ventricles. One patient underwent mapping in both ventricles, and ablation in RV only. Table 3 summarizes the procedural characteristics.

**Table 3:**
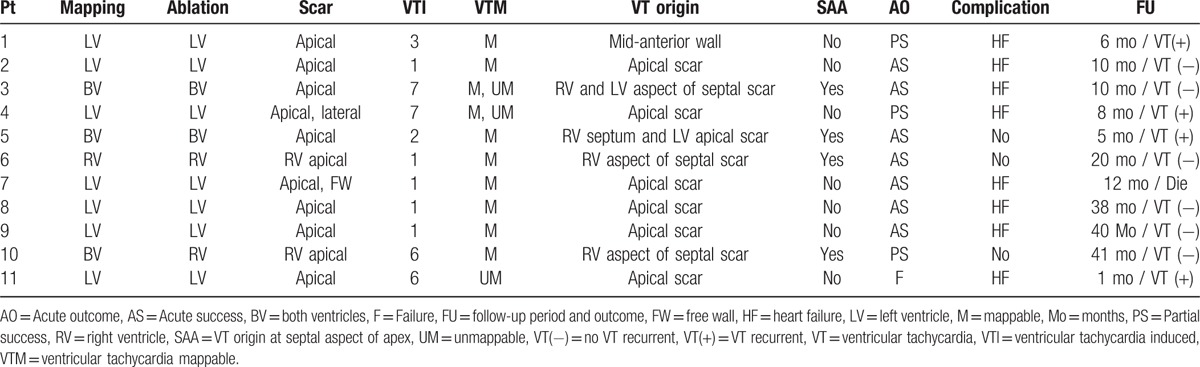
Procedural characteristics and outcomes of patients presenting with left ventricular aneurysm.

**Figure 2:**
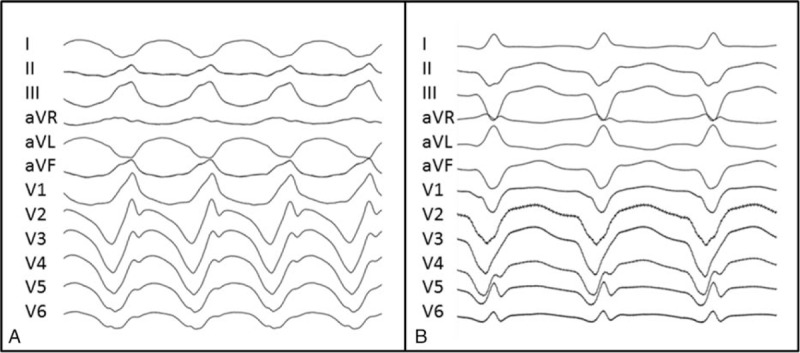
(A) Surface ECG of the first ventricular tachycardia (VT) induced in patient #5 (cycle length: 290 ms). (B) A second type of VT induced (cycle length: 440 ms) in the same patient shows a LBBB pattern.

At electrophysiological evaluation, 36 episodes of sustained monomorphic scar-mediated VT were induced and targeted for ablation in the LVA group. More than one VT was induced in 6 (54.5%) patients (Fig. 2). The mean number of VTs induced per patient was 3.2 ± 2.6 (range 1–7). Ten patients had hemodynamically stable and mappable VTs, 2 of which also had unmappable VTs. One patient only had hemodynamically unstable and unmappable VTs. VTs were related to LVA in 10 patients, and unrelated to LVA in 1 patient (patient #1).

The ablation procedure was successful (noninducibility of any VT after ablation) in 25 (75.7%) patients, partially successful (elimination of only the clinical VT) in 6 (18.2%) patients, and failed in 2 (6.1%).

Complete success was achieved in 7 (63.6%) patients in LVA group and in 18 (81.8%) patients in none LVA group. Partial success was achieved in 3 (27.3%) patients in the LVA group and in 3 (13.6%) patients in none LVA group, while the procedure was a complete failure in 1 patient in each group. The distribution of acute results did not differ significantly between patients with and without LVA (*P* = 0.517).

**Figure 3:**
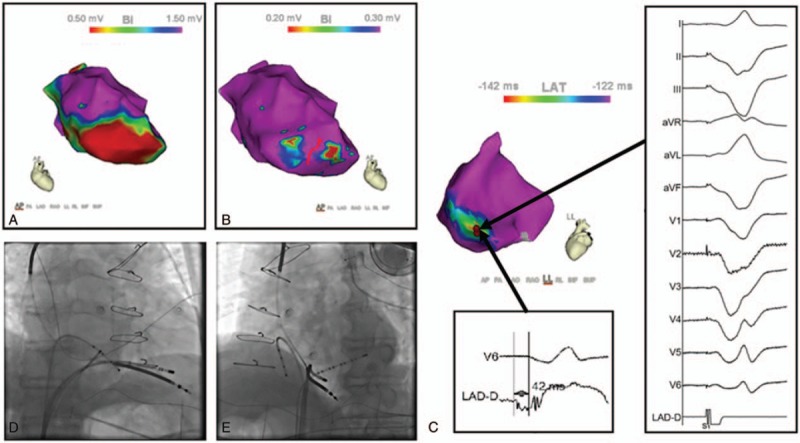
(A) Sinus rhythm voltage map of patient #5. The score scale is shown in the right upper panel. Lowest-amplitude areas are shown as red, progressing to greater-amplitude areas indicated by yellow, green, blue, and purple (amplitude 1.5 mV). (B) With adjustment of voltage cutoff (0.20/0.30 mV), a “channel” is identified transecting the scar. Ablation was targeted at the “channel,” and clinical VT (the first VT in Fig. 2 was eliminated). (C) Recording at the earliest activation site (-42 ms) during sustained VT (LBBB pattern VT in Fig. 2). Pacing at this site revealed a good pace map (defined by matching QRS morphologies when pacing in sinus rhythm and during VT), suggesting that the ablation catheter was localized in a potential isthmus region of the LBBB pattern VT circuit. Fluoroscopy RAO view (D) and LAO view (E), showing the ablation catheter at the right ventricular aspect of septum at the level of the earliest ventricular activation (LBBB pattern VT in Fig. 2). LAO = left anterior oblique, RAO = right anterior oblique, VT = ventricular tachycardia.

As summarized in Table 3, clinical VTs located in apical part of the septum scar zone in 4 (36.4%) patients in the LVA group (Fig. 3). Among the 10 patients who underwent endocardial LV mapping, the reentrant common pathway was targeted in the LV apex in 9 (patients #2, 3, 4, 5, 7, 8, 9, 10, 11). The clinical VTs were located in the septal-apex scar zone in 3 patients (#3, #5, #10). Ablation success of noninducibility of any VT was achieved by targeting abnormal electrograms in right and left aspects of septal scar in patient #3. In patient #4 and #10, a nonclinical VT originated from the septum was still inducible at the end of ablation, and was terminated by burst stimulation. The complete ablation success was achieved by targeting apical scar border zones and abnormal electrograms in the rest 4 patients (patients #2, 7, 8, 9). With hemodynamically unstable VTs were induced, ablation was performed on the basis of substrate and pace-mapping and the clinical VT was still present at the end of ablation in patient #11. VT origin was localized to the RV aspect of septum and was abolished in RV in patient #6. In patient #1, a nonclinical VT originating from the mid-anterior wall was still inducible at the end of ablation.

### 3.3 Procedural complications

Overall, mild worsening of heart failure was recognized in 11 (33.3%) patients, and was relieved by low-dose diuretics. Compared with patients without LVA, patients with LVA were more likely to present worsening heart failure (72.7% vs 13.6%, *P* = 0.001). No other major complications were observed during and after procedures in all patients.

### 3.4 Long-term outcomes

**Figure 4:**
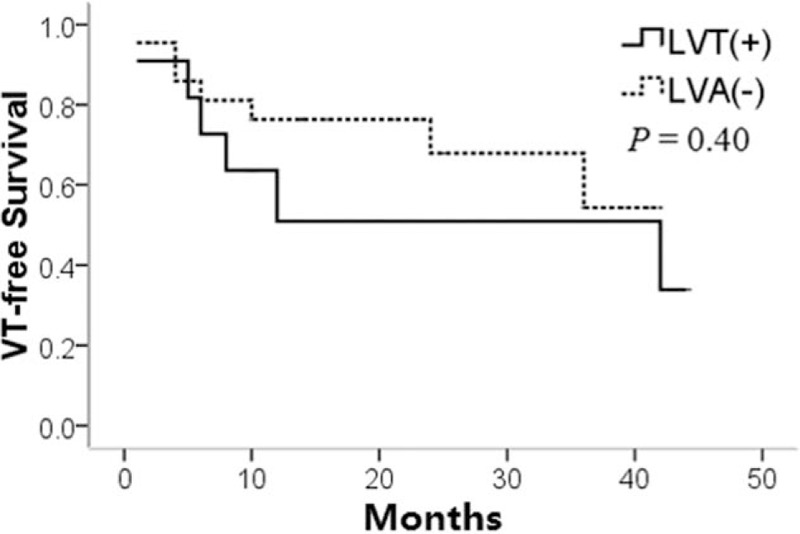
Cumulative VT-free survival for patients with left ventricular aneurysm (continuous line) or without left ventricular aneurysm (dashed line), showing no significant difference between the 2 groups (Log-rank test).

After median 19 (1–44) months follow-up, overall VT-free survival rate was 60.6%. During follow-up, 1 (9.1%) patient died from progressive heart failure 12 months after the initial procedure, and 5 (45.5%) patients developed 1 or more episodes of a sustained VT in the LVA group. As shown in Fig. 4, there was a numerically but not significantly increased prevalence of VT recurrence in patients with LVA compared with patients without LVA (*P* = 0.40). VT-free survival was 48.5% in patients with LVA and 68.2% in patients without LVA during the follow-up.

## 4 Discussion

The main findings of the present study on safety and efficacy of ischemic VT ablation in patients with LVA were as follows: first, LVA was found in about one-third of patients who underwent ablation of post-infarction VT, and patients with LVA were more likely to have impaired cardiac function; second, ablation of VT in the presence of LVA appeared feasible and associated with a similar success rate than in patients without LVA; third, VT ablation in patients with LVA had an acceptable risk of complications among high-risk patients with electrical storm or recurrent ICD shocks; and fourth, over one-third of patients with LVA had septal targets.

LVA is an important complication associated with MI that is related to adverse outcomes, such as lethal arrhythmia, heart failure, and thrombus formation.^[23–26]^ VT is a common and a negative prognostic factor in patients with post-infarction ventricular dilatation and aneurysm. This is of particular importance in patients with endocardial scarring following MI.

RFCA is a safe and effective treatment option for scar-mediated VT.^[6,22,27–30]^ In the randomized Substrate Mapping and Ablation in Sinus Rhythm to Halt Ventricular Tachycardia (SMASH-VT) trial, catheter ablation reduced ICD therapies by 65% during 2-year follow-up in post-MI patients receiving ICDs for secondary prevention.^[28]^ The Ventricular Tachycardia Ablation in Coronary Heart Disease (VTACH) study randomized 107 post-MI patients with stable VT to ICD implantation and VT ablation or ICD implantation alone.^[29]^ At 2 years, VT-free survival was 47% in the ablation group and 29% in the control group. In the present study, with median 19 months follow-up, VT-free survival was 60.6% overall, comparing favorably with prior studies; however, LVA was not specifically considered in SMASH-VT trial and VTACH study.

LVA is not uncommon in patients who undergo ablation of post-infarction VTs, and found on echocardiogram in about one-third of patients in the present study. It is considered as a predictor of poor prognosis. VT ablation in the presence of LVA is challenging because the aneurismal area wall is thin and consists almost entirely of hyaline fibrosis tissue. There is a paucity of data on safety and efficacy of catheter ablation for VT in patients with LVA. In the current study, ablation of VTs in the presence of LVA appeared feasible and associated with a similar success rate than in patients without LVA. Interestingly, a recent study shown that LVA was not associated with a higher rate of recurrent VAs or death [hazard ratio (HR) 1.22 (0.72–2.05), *P* = 0.46] in coronary artery disease patient with ventricular arrhythmias.^[31]^ Furthermore, ventricular arrhythmia is the leading cause of death in LVA patients,^[4]^ thus catheter ablation may have a potential role in improving outcomes in LVA patients.

All patients underwent mapping and ablation in only the endocardium in the current study. Sarkozy et al^[32]^ demonstrated epicardial VT circuits in 6% of patients referred for catheter ablation of recurrent VT, with LVA being less frequent among patients with epicardial circuits.

Monomorphic VT can be induced in the presence of arrhythmogenic substrate such as a LVA, which also can be excised by surgical aneurysm resection. However, a report indicated a high incidence of sudden death (37% of 19 deaths during median 3.7 years follow-up) late after anterior LVA repair if concomitant antiarrhythmic surgery was not performed.^[5]^ Surgical ablation combined with aneurysm resection and myocardial revascularization therefore may be a choice for a selected population of patients.

Sartipy et al^[33]^ presented 53 patients who underwent the Dor procedure with nonelectrophysiologically guided subtotal endocardectomy and cryoablation for VT at the transition zone of the scar and viable tissue. Early mortality was 3.8%; overall survival was 94%, 80%, and 59% at 1, 3, and 5 years, respectively; and freedom from spontaneous VT at mean 3.7 ± 2.0 years follow-up was 90%.

The goal of the Dor procedure is to achieve complete coronary revascularization, reduce LV volume, and restore LV shape to relieve ischemia and reduce wall tension. The study published by Sartipy et al^[33]^ could not bring clarification about which of these components was most effective for each problem. Almost all patients (52/53) underwent CABG in the study by Sartipy et al^[33]^; however, in the present study, there was no indication for revascularization in patients with LVA, who were more likely to have advanced congestive heart failure and CKD, which may increase surgical risks. Thus, the benefit of the Dor procedure for preventing postoperative ventricular arrhythmia in patients in the present study is not concrete.

Furthermore, in the present study, more than one-third of patients with LVA had septal targets, which may be a limitation to surgical repair. In previous studies, more than half the patients with septal MI had right ventricular septal areas that are critical for post-infarction VT and cannot be eliminated by left ventricular ablation alone. ^[34]^

## 5 Limitations

This is a relatively small series of high-risk patients predominantly with VT. Further data are needed to assess the feasibility and safety of VT ablation in a larger cohort of patients with LVA. In addition, a competing risks model for VT recurrence was not used and there may be confounding variables that were not apparent because statistical model building was not performed.

## 6 Conclusions

This study indicates that ablation of VT in the presence of LVA could achieve relatively high efficacy and acceptable complication rates in high-risk patients. Furthermore, more than one-third of the patients with LVA have septal targets for post-infarction VT.
